# Topology Challenge for the Assessment of Living Cell Deposits with Shear Bulk Acoustic Biosensor

**DOI:** 10.3390/nano10102079

**Published:** 2020-10-21

**Authors:** Aleksandr Oseev, Nikolay Mukhin, Céline Elie-Caille, Wilfrid Boireau, Ralf Lucklum, Thomas Lecompte, Fabien Remy-Martin, Jean-François Manceau, Franck Chollet, Thérèse Leblois

**Affiliations:** 1 FEMTO-ST Institute, CNRS UMR-6174, University Bourgogne Franche-Comté, 25000 Besançon, France; celine.caille@femto-st.fr (C.E.-C.); wboireau@femto-st.fr (W.B.); fabien.remy@femto-st.fr (F.R.-M.); jfmanceau@femto-st.fr (J.-F.M.); franck.chollet@femto-st.fr (F.C.); 2 Institute for Micro and Sensor Systems, Otto-von-Guericke-University Magdeburg, 39106 Magdeburg, Germany; nikolay.mukhin@ovgu.de (N.M.); ralf.lucklum@ovgu.de (R.L.); 3 Geneva Platelet Group, Faculty of Medicine, Geneva University, 1205 Geneva, Switzerland; ThomasPierre.Lecompte@hcuge.ch; 4 Haemostasis Unit, Department of Medical Specialities, University Hospital Geneva (HUG), 1205 Geneva, Switzerland

**Keywords:** acoustic biosensor, QCM, platelet, cells surface topology

## Abstract

Shear bulk acoustic type of resonant biosensors, such as the quartz crystal microbalance (QCM), give access to label-free in-liquid analysis of surface interactions. The general understanding of the sensing principles was inherited from past developments in biofilms measurements and applied to cells while keeping the same basic assumptions. Thus, the biosensor readouts are still quite often described using ‘mass’ related terminology. This contribution aims to show that assessment of cell deposits with acoustic biosensors requires a deep understanding of the sensor transduction mechanism. More specifically, the cell deposits should be considered as a structured viscoelastic load and the sensor response depends on both material and topological parameters of the deposits. This shifts the paradigm of acoustic biosensor away from the classical mass loading perspective. As a proof of the concept, we recorded QCM frequency shifts caused by blood platelet deposits on a collagen surface under different rheological conditions and observed the final deposit shape with atomic force microscopy (AFM). The results vividly demonstrate that the frequency shift is highly impacted by the platelet topology on the bio-interface. We support our findings with numerical simulations of viscoelastic unstructured and structured loads in liquid. Both experimental and theoretical studies underline the complexity behind the frequency shift interpretation when acoustic biosensing is used with cell deposits.

## 1. Introduction

The acoustic biosensor approach is a widespread technology for label-free assessment of molecular interactions at biointerfaces. Among them, the shear bulk acoustic resonators are commonly used for biosensing due to their low in-liquid radiation losses. Quartz crystal microbalance (QCM) is a recognized technology that is used to detect interactions at the surface. When the QCM is excited at a shear bulk vibration mode, any resonant frequency shift reflects a change in the acoustic load at the QCM surface. QCM has been introduced by Sauerbrey, who studied the change of resonant frequency in-air as a function of the mass of a rigid solid material deposited onto the QCM surface [1], corresponding to the so called gravimetric regime. Sauerbrey’s approximation is basically a relation between the attached mass and the mass of quartz crystal per unit area, typically presented as

(1) $$\Delta f_{m}=-\frac{2f_{0}^{2}}{\sqrt{\rho_{Q}\mu_{Q}}}\frac{\Delta m}{A}\;,$$

where *f*_0_ is the resonant frequency of the QCM; $\rho_{Q}$ is the density and $\mu_{Q}$ the shear modulus of quartz; Δ*m* is the probed mass attached to the active surface *A*.

Later work of Kanazawa and Gordon [2] has shown that a Newtonian liquid in contact with a QCM creates an acoustic load of the resonator whose equivalent mass is proportional to the density of the fluid and to *δ*, the penetration depth of the shear evanescent wave in the liquid, expressed as

(2) $$\delta =\sqrt{\frac{\eta_{L}}{\pi f_{0}\rho_{L}}}$$

where *ρ_L_* and *η_L_* are the density and the viscosity of the liquid. The resulting frequency shift is

(3) ΔfL=−f032ρLηLπρQμQ

A typical value of the shear wave penetration depth in water at a fundamental vibration mode around 5 MHz is about 250 nm causing a frequency decrease of roughly 2 kHz. If a more viscous liquid, particularly whole blood, replaces water, the penetration depth of the acoustic wave, as well as the frequency shift, increases.

If a rigid layer at the sensor surface is covered by a liquid, the contribution of the layer Equation (1) and the liquid Equation (3) are additive. However, in biosensor applications, the material at the sensor interface is generally viscoelastic and can no longer be approximated as a rigidly attached mass. Viscoelasticity is a frequency-dependent phenomenon correlated with relaxation times of the biomolecule or its segments [3]. A viscoelastic layer simultaneously exposes elastic loading and viscous losses that causes significant deviation of frequency shift from Sauerbrey’s approximation. In this case, the QCM is operating in non-gravimetric regime [4] and responds to the viscoelastic properties of the layer [5].

Acoustic load of complex layer arrangement have been described by using acoustic impedance concept [6,7]. The surface acoustic impedance represents the overall acoustic load at the interface and is given by

(4) $$Z_{L}=jMV$$

where M=ωρh is the mass factor (*ρ* is the density and *h* is the thickness of the load layer) and V=tanφ/φ is the acoustic factor with *ϕ* being the phase shift of the acoustic wave in the layer. For V=1  we are operating in the gravimetric regime, but it is worth to note that V<1 even for very thin (bio)films in contact with a liquid and appears as a “missing mass” [8,9,10,11].

While the QCM frequency shift for monolayer loading is well described, the theory still lacks sufficient background for inhomogeneous loading when both liquid and solid/viscoelastic materials are in contact with the sensor surface. Moreover, the influence of dimensional effects on the overall frequency shift is rarely discussed. Martin et al. [12] partly addressed the issue considering two-dimensional solid structures and showed that the liquid trapped in the structure contributes with an additional loading.

The assessment of cell deposits with shear bulk acoustic approach should take into an account all the above-mentioned contributions. However, they are often neglected and the QCM frequency shift is commonly attributed to ‘mass loading’ only. Some of the challenges associated with living cell assessment using acoustic biosensor approach were already addressed. It was shown that for a typical mammalian cell with dimensions of 10 μm, only a small part of the cell leads to a frequency shift [13]. Cells deposited onto the surface without firm attachment via a bio-chemical bond can create a liquid filled region between the cell and the vibrating surface. The sensor response at different stages of the cell–surface interaction reflects the nature of the cell attachment, its structural modification, secretion, and expression of extracellular vesicles and its surface spreading [14]. The cell-biointerface interaction triggers cellular biophysical mechanisms that in turn affect its material properties and vary the frequency shift [15]. Actually, QCM frequency shift has been previously related to the spreading of the attached cells [13]. The QCM dissipation factor was also shown to provide a distinctive response to the cell spreading at extremely low cell coverage, when the frequency shift is no longer detectable [16]. Cell stiffness and the changes in the cytoskeleton were reported to predominantly contribute to this frequency shift [17], but viscosity contributions were not clearly identified.

Our work is focused on the study of platelet deposits onto a relevant reactive biointerface for the study of the physiological process of primary hemostasis [18]. Platelets are small discoid shaped anucleated blood cells of 2–3 µm in largest diameter, which circulate in resting state in blood [19]. Their primary function is to prevent and stop bleeding at the site of an injured vessel, thanks to a sequence of interactions between the vessel wall, blood proteins, and platelets, culminating in a platelet plug [20]. The surface coverage with platelets can vary widely from localized (low coverage) to spread (high coverage) deposits, which could affect the acoustic loading conditions. Since most of cell studies are conducted in liquid environment, the acoustic shear vibrations excite evanescent waves that are partially distributed in the viscoelastic biolayer and in the liquid. One finally must consider that some liquid may be trapped between the cells, whereas parts of the cells are separated from the biointerface by a thin liquid layer. The analysis of such platelet deposits with acoustic sensors thus becomes a multifactorial problem [21].

In the following contribution, we concentrate on the assessment of topologically different platelet deposits. We challenge the idea that the frequency shift is self-sufficient to assess topologically different cell deposits. We show the results of QCM biosensor measurements for different types of platelet deposit topologies. We address how the biosensor frequency shift is impacted by platelet deposits topology at the QCM surface and for which condition the frequency shift is directly related to the amount of deposited cells. The results of our numerical studies explain how the deposit topology at the surface impacts the frequency shift response of the QCM biosensor.

## 2. Materials and Methods

### 2.1. Materials

QCM crystals of 14 mm diameter with fundamental frequency 5 MHz (QCM5140CrAu120-050-Q, Quartz Pro AB, Stockholm, Sweden) were utilized for the experimental studies as standard shear bulk acoustic resonant sensors. Each crystal was manually prepared with a collagen biointerface and assembled into a microfluidic cell for blood perfusion tests. Collagen Horm^®^ (type I) and isotonic glucose solution pH 2.7–2.9 (SKF solution) (Takeda Austria GmbH, Linz, Austria) were used for this procedure. N-(3-dimethylamino-propyl)-N-ethylcarbodiimide (EDC), N-hydroxysulfosuccinimide (sulfo-NHS), phosphate buffered saline (PBS), bovine serum albumin (BSA), ethanolamine HCl 1M, 16-mercaptohexadecanoic acid (16-MHA), and 11-mercapto-1-undecanol (11-MUOH) (Sigma Aldrich, Lyon, France) were used as solutions for the biointerface processing. Fixation of cell deposits after each experiment was done with glutaraldehyde 0.5% concentration solution in deionized water (Sigma Aldrich, France). The grafting of collagen followed a procedure established previously for protein grafting on gold chip in our research group [22].

Whole blood samples from healthy male donors were provided by the Etablissement Français du Sang (EFS, Besancon, France), in compliance with current ethical regulations in France. Whole blood was collected into 1.6 mL collecting tubes with hirudin as anticoagulant and used for the experiments within two hours.

### 2.2. Collagen Biointerface

Before engaging experimental investigations in whole blood with rheological conditions that are relevant to microcirculation, it is crucial to control the supramolecular architecture of the biointerface. We used a collagen type I layer to initiate platelet adhesion, activation and plug growth via interaction with additional platelets. The reconstitution of the collagen layer is performed on a self-assembled monolayer of 16-MHA and 11-MUOH onto the gold surface of the chip; this ensures a covalent grafting of collagen and a robustness under the shear rates used in this study. An in-depth characterization of that collagen biointerface included the surface coverage, topological profiles, the thickness, and orientation of the collagen fibers.

**Figure 1:**
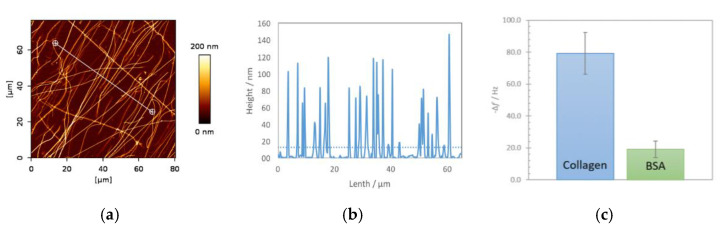
AFM images of immobilized fibrillar collagen type I on QCM sensing interface, height trace (**a**) and height profile with an average height for the whole scanned surface shown as dotted line (**b**). Measurements of QCM frequency shift caused by immobilized collagen biointerface and passivating BSA as a negative control (three times repeated measurements) (**c**).

AFM image of immobilized collagen on gold surface, its height measurement profile and corresponding frequency shift (including negative control with BSA) are shown in Figure 1.

Reproducibility of the grafting is a prerequisite for sensing purposes, especially in terms of covering rates which are of 23% ± 2.3% (based on AFM images) in this architecture with a mean collagen fiber height of 86 ± 12 nm. The assessment of the acoustic load imposed by the biointerface itself, in-air condition, was completed using several QCM sensors. Measurements were performed before and after the collagen immobilization revealing an average shift close to 80 Hz. As a negative control, reference QCMs were processed within a similar immobilization protocol omitting the step of collagen immobilization. They presented an average shift close to 20 Hz, likely to represent the loading caused by passivating BSA protein. The results of recorded frequency shift in both cases are shown in Figure 1c and demonstrated a standard deviation below 10%.

### 2.3. Experimental Setup and Conditions

**Figure 2:**
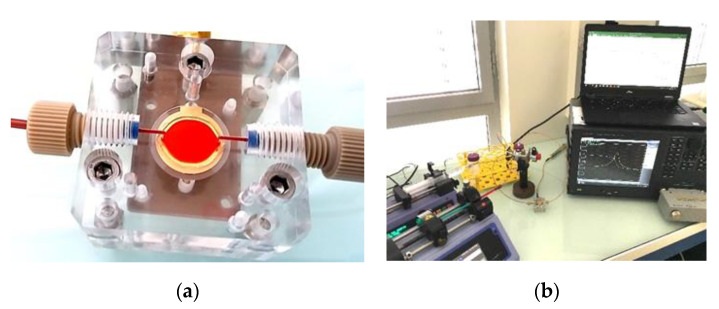
Photograph of the whole blood perfusion chamber with an installed QCM biosensor (**a**) and the experimental setup (**b**).

A Poly(methyl methacrylate) transparent microfluidic chamber of 50 µm height with the collagen coated QCM defining the floor plane was fabricated to mimic experimental conditions similar to microcirculation. Perfusion chamber and experimental setup are shown in Figure 2.

The whole blood perfusion through the chamber was performed for 5 min at room temperature, which was kept stable in the range 23–24 °C. The perfusion tests were made at rheological conditions corresponding to small vessels by using microfluidic setup with separate injection lines [23]. The QCM admittance spectrum was continuously recorded during the blood perfusion with a Keysight 4990A (Keysight Technologies, Santa Rosa, CA, USA) impedance analyzer using custom made software. This spectrum is used to track the evolution of the QCM resonant frequency. The ‘frequency shift’ used in the experiment is referenced to the resonance frequency obtained when the biointerface is in contact with the PBS buffer (zero frequency shift).

The deposited cells on the QCM were subsequently studied with Nanowizard III AFM (JPK Instruments, Berlin, Germany) in order to characterize the surface topography of the biointerface. Images were taken for the maximal scanning area 100 × 100 µm using Nano World NPS-10C cantilevers (NanoAndMore, Paris, France) made from silicon nitride with a stiffness of 0.32 N/m. The cellular deposits were fixed with 0.5% solution of glutaraldehyde in deionized water. The images were obtained in a contact mode in air at a frequency of 0.5 line/s with a resolution of 512 × 512 pixels. Height trace scans were recorded to evaluate the amount and morphology of platelet deposits. The average thickness of cells deposits was determined by an arithmetic mean value of the height across the 100 × 100 µm scanned area. The coverage was measured as a ratio of the surface covered by platelet deposits to the whole scanned surface when 0 corresponds to free surface and 1 to full surface coverage.

### 2.4. Platelet Deposits Characterization

**Figure 3:**
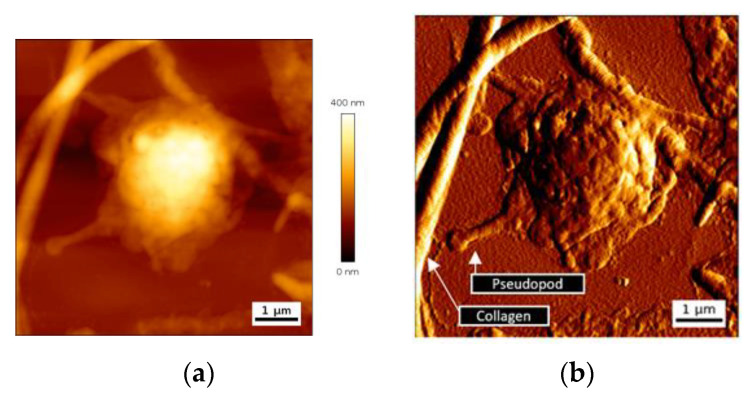
AFM image of a single platelet attached to collagen fibers, height trace (**a**) and deflection trace (**b**).

Platelet-collagen interaction leads to firm platelet adhesion. Structural modifications of platelets ensue, with cytoskeleton modifications, release of granule content, and cell spreading. We have first looked at single platelet deposits to assess their dimension and more particularly their thickness. AFM images of height and deflection traces for a representative single adherent platelet on collagen biointerface are shown in Figure 3.

Adherent single platelets have a semispherical shape with a variable spreading. Deflection trace image reveals pseudopods emitted by cells and the presence of nanoscale vesicles close to the cells, and probably released by them (Figure 3b). Maximal height of a single deposited platelet was in a range 460 ± 70 nm.

**Figure 4:**
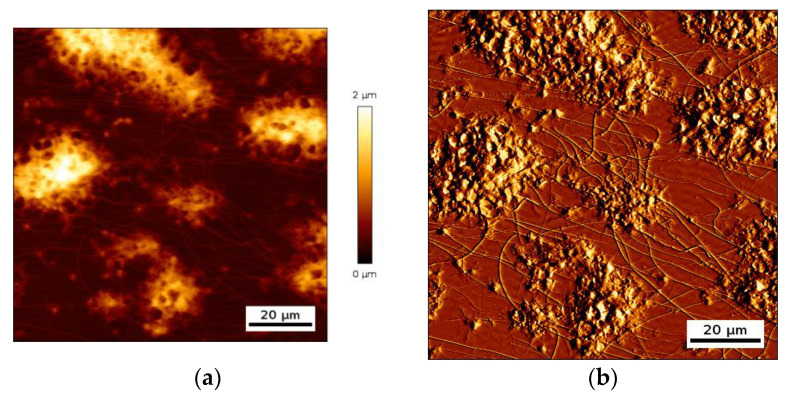
AFM images, height trace (**a**) and deflection trace (**b**), of multiple platelet deposits forming an aggregated state on the collagen biointerface.

Interaction of platelet GPVI receptor with collagen leads to activation of platelet α_IIb_β_3_ integrin, which enables the direct interaction of deposited platelet with circulating ones [24,25,26]. As a result, platelets aggregate and form deposits of varying topology. The deposits are distributed across the biointerface with lateral dimensions that are nearly two orders of magnitude larger than the height of a single adherent platelet [27,28]. An example of a deposit of aggregated platelets is shown in Figure 4.

One can clearly distinguish single platelet deposits from platelet aggregates with a lateral dimension of several tens of micrometers and a maximal height in some cases reaching two micrometer. Those platelet deposits create a complex surface topology, with some areas of the biointerface that are completely covered with platelets and others that are directly in contact with the liquid, which is whole blood during such an experiment. As a result, the acoustic resonator surface sees a distributed cells/liquid load.

### 2.5. Computational Model

**Figure 5:**
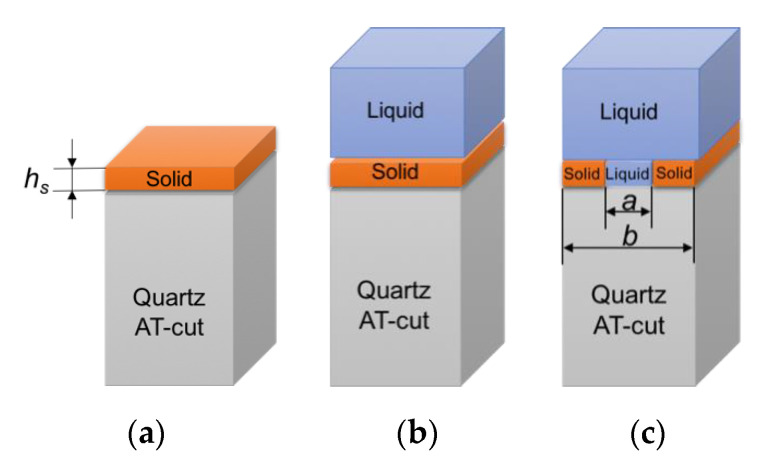
Schematic representation of computational model of quartz resonator loaded with solid monolayer of thickness *h_s_* in-air (**a**), in-liquid (**b**); solid structured layer in-liquid with period *b* and aperture *a* (**c**).

Numerical studies were performed with COMSOL Multiphysics 5.4 software (COMSOL France, Grenoble, France). A three-dimensional computation model with periodic boundary conditions in X and Y directions was developed. The acoustic resonator was described as a piezoelectric domain with AT-cut quartz of 330 μm thickness. The solid layer load was simulated with a structural mechanics model. Solid fluid interactions were computed with the acoustic-structure interaction model. We used two different liquids, the buffer liquid (Liquid1—density 1000 kg/m^3^; viscosity 0.001 Pa·s) and the whole blood (Liquid2—density 1060 kg/m^3^; viscosity 0.003Pa·s). Although whole blood is a non-Newtonian fluid (shear thinning effect), above a shear rate of 200 s^−1^, as used in this study, its viscosity may be considered constant [29]. The validation of numerical model was completed for the computational problems that are schematically shown in Figure 5.

**Figure 6:**
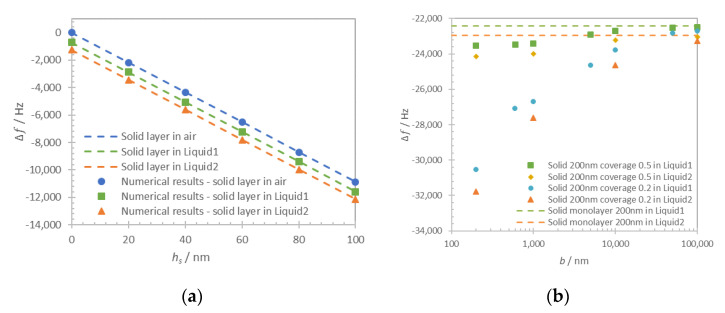
(**a**) Results of numerical computation (markers) and analytical approximation (dashed lines) of acoustic resonator loaded with a solid layer made of gold in a thickness range 0–100 nm in air and in-liquid for Liquid1 and Liquid2. (**b**) Computational results for structured solid layer with average thickness 200 nm under liquid loading: Liquid1 (buffer equivalent) coverage 0.2 (blue circles) and 0.5 (green squares), Liquid2 (blood equivalent) coverage 0.2 (red triangles) and 0.5 (orange diamond). Analytical approximation for a solid monolayer of 200 nm thickness under liquid loading for Liquid1 (green dashed line) and Liquid2 (orange dashed line).

The thickness of the solid layer (*h_s_*) is varied in a range 0–100 nm with a step of 20 nm. Computational results for frequency shift of maximum of admittance for the solid monolayer in-air and in-liquid for Liquid1 and Liquid2 are shown in Figure 6a (dots). The respective approximations with Equation (1) for a solid layer in-air and the sum of Equations (1) and (3) for the case in-liquid are shown in Figure 6a as dashed lines. Zero frequency shift corresponds to the unloaded resonator in air.

In contrast to the monolayer type of loading, the structured solid layer presents more complex behavior. In this case, the resonator sensing surface is partially covered with a solid layer and in a partial contact with the liquid. The solid layer was defined as a two-dimensional arrangement of rigidly attached lines with a variable period *b* in a range 0.2–100 μm. In fact, the term period is used to simplify the computation problem only with no claimed effects that depend on the existence of a periodicity. The coverage Cov  was defined as the ratio between the surface covered by the solid layer and the total surface: $Cov=\frac{b-a}{b}$. Two representative values of coverage 0.2 and 0.5 were used in this computation. The average thickness of the structured solid layer $\bar{h}=Cov*h_{s}$ was always kept equal to 200 nm to ensure similar solid mass loading contribution in each case. The thickness of the solid layer was then respectively defined as 400 nm for coverage 0.5 and 1 μm for coverage 0.2. The computation was performed for Liquid1 and Liquid2 and the results are shown in Figure 6b.

As it can be seen in Figure 6a, the numerical results and analytical approximations for continuous film loading of quartz resonator are in close agreement (discrepancy lower than 0.2%), proving the validity of the developed computational model. In the case of both liquid and solid monolayer loadings, the additive contribution of rigidly attached solid Equation (1) and liquid Equation (3) clearly cause the shift of the resonant frequency.

For the discontinuous film in Figure 6b, we distinguish two different loading regimes that are regulated by the period of the layer structures. When the period is several times larger than the layer thickness, the approximation of the loading can be made using the additive contribution of Equations (1) and (3). When the period is comparable or smaller than the thickness of the layer this approximation is no longer valid, particularly in the low coverage case (*Cov* = 0.2). The frequency shift in this case is impacted by the contribution of trapped liquid and can be obtained only numerically.

## 3. Results

### 3.1. Experimental Results

**Figure 7:**
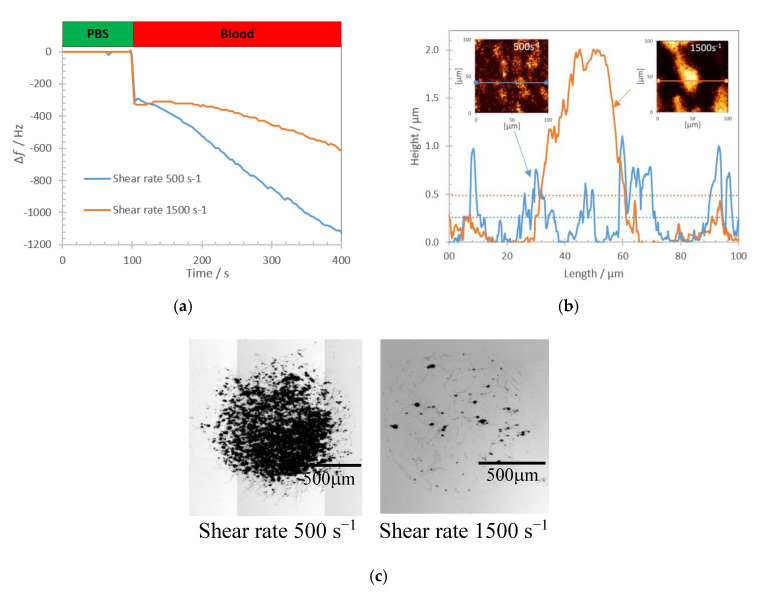
(**a**) Real-time recorded frequency shift during 5 min of whole blood perfusion at wall shear rates 500 s^−1^ and 1500 s^−1^. (**b**) Height profile of platelet deposits across the biointerface for shear rates 500 s^−1^ (blue curve) and 1500 s^−1^ (orange curve) (average height for 500 s^−1^ (blue) and 1500 s^−1^ (orange) are shown with dotted lines). Insets: AFM images (height trace) of platelet deposits obtained at 500 s^−1^ (on the left) and 1500 s^−1^ (on the right) shear rates respectively. (**c**) Optical microscopy image of platelets deposits on collagen biointerface at 500 s^−1^ (on the left) and 1500 s^−1^ (on the right).

The experimental study was focused on the evaluation of platelet deposits of different topology that were obtained by varying the wall shear rate condition of perfused whole blood. Shear rate influences platelet adhesion and growth of aggregates. We have performed the experiments in a range of shear rates 500–1500 s^−1^ that is relevant to the microcirculation. Real time changes of the QCM frequency shift were recorded during the whole blood perfusion for two different cases corresponding to the wall shear rates 500 s^−1^ and 1500 s^−1^ and are shown in Figure 7a. Zero frequency shift corresponds to the state when QCM is loaded with the biointerface in PBS buffer.

Perfusion of whole blood causes a rapid frequency downshift caused by the change of viscosity and density of the medium at the sensor interface (change from PBS to whole blood). Subsequent frequency shift can be attributed to the deposited blood material, predominantly blood platelets as evidenced on AFM images. The images and the corresponding height profiles of two extreme cases with the wall shear rate 500 s^−1^ and 1500 s^−1^ are shown in Figure 7b. As can be seen, platelets may form evenly distributed or localized deposits depending on the applied shear conditions. When compared to a single platelet thickness, the height of the platelet deposits suggest that they consist of several platelets stacked together.

**Figure 8:**
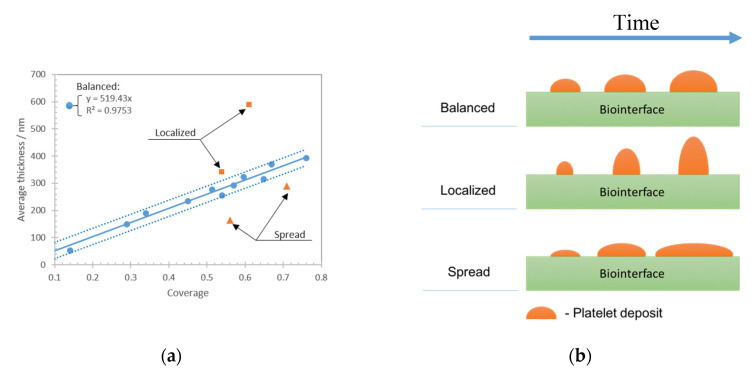
Platelet deposits average thickness versus deposits coverage. (**a**) Blue dots show the cases that are found within one standard deviation (dashed lines) of the linear regression (solid line). Orange dots correspond to the cases with deviation from linear regression larger than one standard deviation. Linear regression (solid line) and corresponding coefficient of determination *R*^2^ are shown for the data points within one standard deviation (blue dots). (**b**) Schematic illustration of the deposits growth for the ‘balanced’, ‘localized’, and ‘spread’ cases.

In the following, we used 500–1500 s^−1^ range of shear rates to conduct experiment with various platelet deposits topology. The kinetics response of the QCM sensor was recorded for each case and used to deduce a frequency shift caused by the deposited cells reduced by negative control. The amount of platelet deposits was evaluated by AFM as an average thickness across a 100 × 100 μm scanned area. The surface coverage was evaluated as a ratio of surface covered with platelets to the whole scanned surface. Figure 8a shows the average thickness versus coverage of the platelet deposits from different experiments that were grouped in three zones named as localized, balanced, and spread. Figure 8b schematically shows these three platelet deposits growth types. The ‘balanced’ case happens when the increase in deposit height is proportional to the increase in coverage. The cases are called ‘localized’ when the growth occurs predominantly in height with minor increase in coverage and, vice versa, they are ‘spread’ when growth occurs predominantly in lateral direction (mostly coverage increase).

We applied linear regression to all obtained data points by using the least squares method. Standard deviation for all obtained coverage/thickness points was calculated as a square root of data variance found as a squared deviation of values from linear regression. The data points that stay within one standard deviation are shown in blue and correspond to the ‘balanced’ condition, and the data points outside one standard deviation are shown in orange. The determination coefficient for the data within standard deviation is *R*^2^ = 0.9753 and for all the data points it is *R*^2^ = 0.5296. The region of the plot above the regression curve was defined as the “localized” and the one below the curve as the ‘spread’ type of platelet deposits. Existence of such linear regression would mean that, at different shear rate conditions, platelets still follow certain growth tendency at which the increase in average thickness of formed platelet deposits is correlated to the increase of coverage. This dependence for the studied conditions is described as a ‘balanced’ growth of platelet deposits. Experimental results that are shown in orange dots represent two different cases of ‘localized’ and ‘spread’ deposits. The cases above the curve correspond to ’localized’ ones where the thickness of platelet deposits is higher than the regression value for the same coverage. That types of deposits were obtained at high shear rate values. The ‘spread’ state of platelets deposits (below the curve) corresponds to the cases when the platelet deposits of similar average thickness show higher coverage. These deposits were obtained at low shear rate conditions.

**Figure 9:**
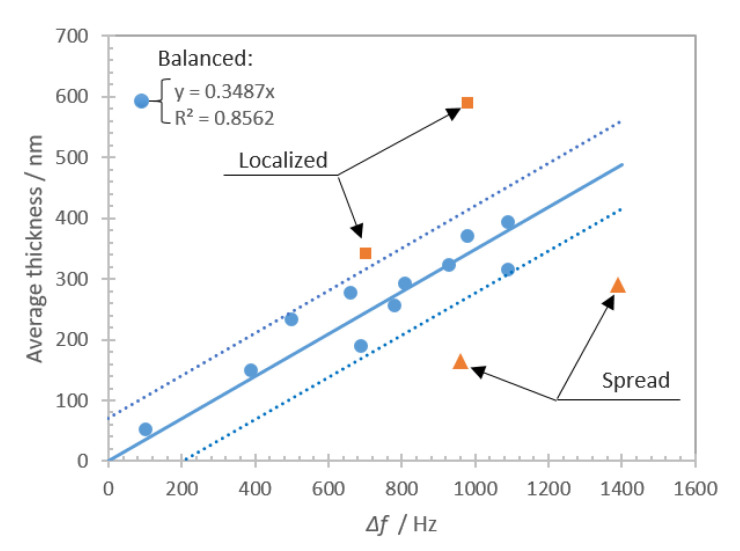
Results of experiments plotted as average thickness of platelet deposits versus frequency shift. Blue dots show the cases that are found within one standard deviation (dashed lines) of the linear regression (solid line). Orange dots are the cases beyond one standard deviation for the linear regression of thickness/coverage data. Determination coefficient *R*^2^ corresponds to the linear regression (solid line) of the data shown with blue dots.

Figure 9 shows the same data points but this time plotting average thickness versus frequency shift reduced by negative control.

The results show that the frequency shift exhibits similar tendency (blue dots *R*^2^ = 0.8562, all the data *R*^2^ = 0.3032) than the average thickness/coverage regression shown in Figure 8. The same ‘localized’ deposits show lower frequency shift for similar average thicknesses while the same ‘spread’ deposits demonstrate a much higher frequency shift for similar average thicknesses.

### 3.2. Theoretical Results

**Figure 10:**
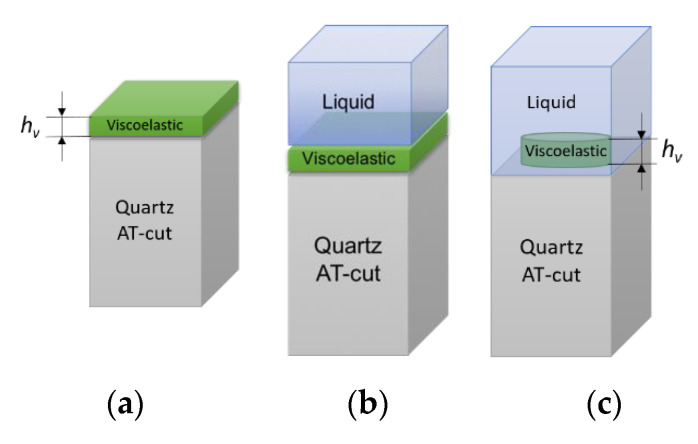
Schematic representation of computational model of QCM loaded with viscoelastic layer in-air (**a**), viscoelastic monolayer in-liquid (**b**); viscoelastic cylindrical structures in-liquid (**c**).

To reproduce cellular viscoelastic behaviour we implemented viscoelastic domain based on the Kelvin–Voigt model. The material parameters were defined in accordance with the range reported for blood platelets, that is the elastic modulus 10 kPa and the viscosity 0.01 Pa·s [30,31]. The computational domains are schematically shown in Figure 10.

**Figure 11:**
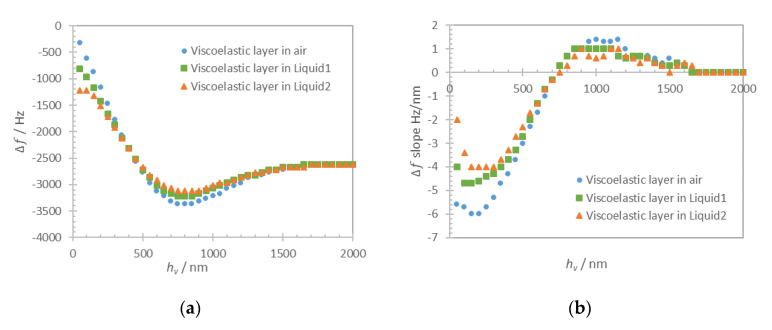
Computation results for the viscoelastic loading of acoustic resonator, frequency shift Δ*f* (**a**) and the slope of the frequency shift (Δ*f* slope) curve (**b**) versus viscoelastic layer thickness (*h_v_*) in-air (blue dots), under Liquid1 (green squares) and Liquid2 (red triangles). The frequency shift is calculated relative to the frequency of unloaded resonator in air.

The thickness of the viscoelastic layer (*h_v_*) was varied in a range 50–3000 nm with steps of 50 nm. The frequency shift of the admittance resonance (Figure 11a) and its derivative (Figure 11b) are plotted versus the thickness of the viscoelastic layer in-air and in-liquid for the two previously defined liquids.

The results in Figure 11a show a gradual frequency downshift for the viscoelastic layer thickness variation up to 700 nm that is larger than what is generally accepted for cells which is 250 nm (shear wave penetration depth in water for QCM 5MHz fundamental mode). For all the studied cases, the frequency shift exhibits a minimum at around 700 nm (slope is zero), followed by an increase of frequency corresponding to an effective “missing mass” [32,33,34]. Finally, the evolution of the frequency shift reaches a saturation at around 1.5 μm of viscoelastic layer thickness.

**Figure 12:**
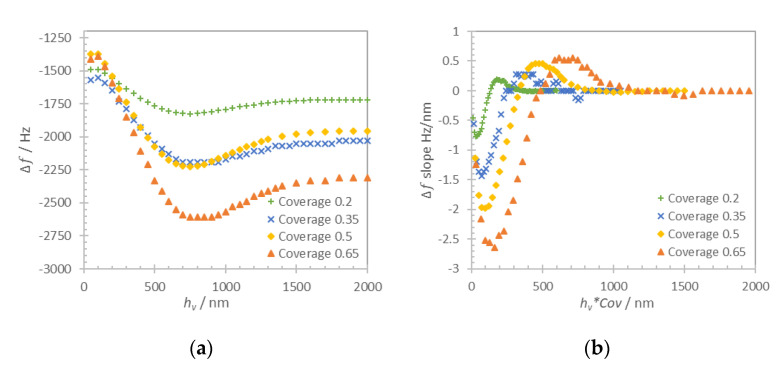
Computation results for acoustic resonator loading with structured viscoelastic layer of period 100 nm with the coverage in a range 0.2–0.65 and a range of thicknesses 50–2000 nm. Frequency shift versus structure thickness (**a**) and slope of frequency shift versus thickness (*h_v_*) multiplied by coverage (*Cov*) for each structured layer (**b**). The frequency shift is calculated relative to the unloaded resonator frequency in air.

The frequency shift of the acoustic resonator loaded with a structured viscoelastic layer was studied for cylindrical viscoelastic pillars with a surface coverage in a 0.2–0.65 range with a step of 0.15. We chose a period of 100 μm for the structured layer that was shown previously to present low liquid trapping effect for the structured solid layer (Figure 6b). Numerical simulation results of the resonator frequency shift versus the thickness of the viscoelastic structure (*h_v_*) are shown in Figure 12a. The slope of the frequency shift as a function of the average structure height (h_v_*Cov) is shown in Figure 12b.

Figure 12a shows that the coverage of the viscoelastic layer affects the range of frequency shift of the acoustic resonator. As expected, the layers with higher coverage result in a larger frequency shift, the saturation of frequency shift being reached at similar thickness of the pillar structure but at a different level of frequency shift. Figure 12b shows the slope of frequency shift depending on the total amount of viscoelastic material that is shown as the thickness of viscoelastic layer multiplied by coverage (h_v_*Cov). As it can be seen, the coverage affects the slope of the frequency shift for the same total amount of viscoelastic material at the surface. In terms of sensing application, the viscoelastic structures with larger coverage would result in higher frequency shifts even though the average height (that is, the total amount of viscoelastic material) is the same.

**Figure 13:**
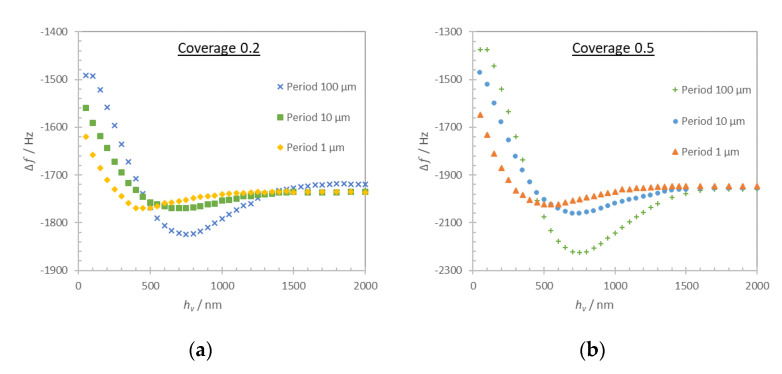
Computation results for acoustic resonator loading with a structured viscoelastic layer of period 100, 10, and 1 µm and coverage 0.2 (**a**) and 0.5 (**b**) versus the structure thickness. The frequency shift is calculated relative to the unloaded resonator frequency in air.

We estimated the effect of the trapped liquid using cylindrical viscoelastic structures of period 100, 10, and 1 µm (Figure 10c). Periods below 1 µm are not considered since the pillar eigenmodes would interfere with the data. We considered two surface coverages, 0.2 and 0.5, and the frequency shift as a function of the cylinder thickness are shown in Figure 13a,b respectively.

The results show that liquid trapping in the viscoelastic structure also contributes to the frequency shift. The reduction of the structure period causes an increase of the frequency shift in the thickness range 50–500 nm. The nonlinear behavior of the response in the thickness range 500–1500 nm is strongly modified with the same tendency for both coverages. The saturation of the frequency shift is obtained at almost the same thickness value (about 1500 nm) for all studied structure periods with minor difference between the two coverage cases.

Summarizing the theoretical results, we see that the surface coverage of the viscoelastic deposits regulates the range and slope of the frequency shift. The dependence of the frequency shift versus the thickness of the viscoelastic structure being different for each coverage case. Moreover, the trapped liquid contributes to the frequency shift, depending on the period of the viscoelastic structure. Therefore, an accurate estimation of the thickness of the viscoelastic deposits using the frequency shift would require the information about its topology. With this knowledge, the frequency shift could be computed numerically for known material properties of viscoelastic medium and liquid. The accurate comparison of the amount of viscoelastic deposits between different cases can be obtained when the topology is unchanged or if it follows a known tendency. Conversely, deposits with various thickness/coverage will show high deviation of frequency shift without necessarily having large difference in deposit thickness. These two scenarios were observed experimentally corresponding, respectively, to the balanced and to the spread/localized cases.

## 4. Discussion

This study was focused on the assessment of living cells, i.e., platelet deposits onto a biologically reactive surface, with an acoustic biosensor. The study consisted of experimental investigations supported with computational results. Before addressing the particular behavior of platelets on the sensing layer, we have deeply characterized the biointerface by various biophysical techniques (QCM, AFM, optical microscopy). Once well established in a reproducible manner, it was possible to extend the investigation to platelets.

The experimental and numerical results show that the effective loading of the shear bulk acoustic resonator with living cells is a challenging problem, as the topology of cell deposits plays a significant role in the overall sensor response. Platelet–biointerface interaction is a dynamic process with a certain equilibrium between the amount of deposited cells on a given area (defined as an average thickness) and overall coverage. We show theoretically that the topology of the viscoelastic layer impacts the sensitivity and the maximum range of the biosensor. This hypothesis can explain the experimental observations for ‘localized’ and ‘spread’ deposits that are clearly distinct from ‘balanced’ cases. It was shown that, for the ‘balanced’ deposit, the biosensor frequency shift approximates accurately the thickness of the platelet deposits. On the other hand, it was found that for “localized’ and ‘spread’ deposits the frequency shift significantly deviates from expected values. The ‘spread’ cells deposits affect the sensor as a ‘monolayer’ type of loading, which theoretically shows the largest frequency shift range and slope. Thus, the ‘spread’ platelet deposits have larger frequency shift for the same average thickness compared to the balanced case. On the other hand, ‘localized’ cells deposits have lower coverage comparing to a monolayer, that leads to a reduction of the frequency shift range and slope. Moreover, the computational results clearly demonstrate the contribution of trapped liquid depending on the period of the structure. For experimentally obtained deposits, that would be an added loading that depends on the morphology of aggregated cells. Summarizing both experimental and theoretical results, it can be stated that an accurate evaluation of the amount of deposited cells using frequency shift measurement requires an evaluation of the topology of the formed cellular deposits. The reason is the multiple contributions from different loading mechanisms that are challenging to decouple from the measured frequency shift of the shear bulk acoustic resonator.

The results presented in this contribution are discussed only in terms of topological effects of cellular deposits and do not consider the complex nature of the cell and its changes of material properties. All these effects are likely to contribute to the frequency shift and should also be carefully studied.

## 5. Conclusions

This work was focused on experimental and theoretical studies of living cells assessment with a shear bulk acoustic resonator biosensor. The study showed that the resonator frequency shift depends not only on the amount of blood platelets deposits but also on their topology. First, the frequency shift was different for different topologies formed by equal average thickness of platelet deposits. This is the result of differences in the slope of frequency shift and in liquid trapping contribution depending on the cell deposits topology at the sensor surface. Second, the deposit coverage controls the range of thicknesses where the sensor remains sensitive to viscoelastic loads. The lower the coverage, the lower the deposit thickness at which the biosensor response reaches saturation. This is directly linked to the constant penetration depth of the acoustic waves in the viscoelastic deposit.

Our studies showed that the frequency shift measurement obtained for different viscoelastic deposits at the sensor surface have to be carefully calibrated with respect to the deposit topologies. It makes the assessment of living cells using an acoustic biosensor alone a very challenging task. In fact, the simple interpretation of the frequency shift as a measure of the amount of cells is prone to errors as the shift depends on cell deposits topology as well.

The herein-presented study was performed with human blood platelets but the results can be extended to other types of cells as soon as they are assessed with a shear bulk acoustic resonator based biosensor.

## References

[1] Sauerbrey G. (1959). Verwendung von Schwingquarzen zur Wägung dünner Schichten und zur Mikrowägung. Z. Phys..

[2] Keiji Kanazawa K., Gordon J.G. (1985). The oscillation frequency of a quartz resonator in contact with liquid. Anal. Chim. Acta.

[3] John D. (1980). Ferry Viscoelastic Properties of Polymers.

[4] Lucklum R., Hauptmann P. (2006). Acoustic microsensors-the challenge behind microgravimetry. Anal. Bioanal. Chem..

[5] Lucklum R., Behling C., Hauptmann P. (1999). Role of mass accumulation and viscoelastic film properties for the response of acoustic-wave-based chemical sensors. Anal. Chem..

[6] Behling C., Lucklum R., Hauptmann P. (1998). The non-gravimetric quartz crystal resonator response and its application for determination of polymer shear modulus. Meas. Sci. Technol..

[7] Lucklum R., Hauptmann P. (2000). Quartz crystal microbalance: Mass sensitivity, viscoelasticity and acoustic amplification. Sens. Actuators B Chem..

[8] Rodahl M., Kasemo B. (1996). On the measurement of thin liquid overlayers with the quartz-crystal microbalance. Sens. Actuators A Phys..

[9] Antonio A. (2008). Piezoelectric Transducers and Applications.

[10] Voinova M.V. (2015). Modelling of the response of acoustic piezoelectric resonators in biosensor applications—Part 1: The general theoretical analysis. J. Sens. Sens. Syst..

[11] Voinova M.V. (2009). On mass loading and dissipation measured with acoustic wave sensors. J. Sens..

[12] Martin S.J., Wessendorf K.O., Gebert C.T., Frye G.C., Cernosek R.W., Casaus L., Mitchell M.A. Measuring liquid properties with smooth- and textured-surface resonators. Proceedings of the Annual Frequency Control Symposium.

[13] Rodahl M., Höök F., Fredriksson C., Keller C.A., Krozer A., Brzezinski P., Voinova M., Kasemo B. (1997). Simultaneous frequency and dissipation factor QCM measurements of biomolecular adsorption and cell adhesion. Faraday Discuss..

[14] Modin C., Stranne A.L., Foss M., Duch M., Justesen J., Chevallier J., Andersen L.K., Hemmersam A.G., Pedersen F.S., Besenbacher F. (2006). QCM-D studies of attachment and differential spreading of pre-osteoblastic cells on Ta and Cr surfaces. Biomaterials.

[15] Wegener J., Seebach J., Janshoff A., Galla H.J. (2000). Analysis of the composite response of shear wave resonators to the attachment of mammalian cells. Biophys. J..

[16] Fredriksson C., Khilman S., Kasemo B., Steel D.M. (1998). In vitro real-time characterization of cell attachment and spreading. J. Mater. Sci. Mater. Med..

[17] Heitmann V., Reiß B., Wegener J. (2007). The Quartz Crystal Microbalance in Cell Biology: Basics and Applications. Piezoelectric Sensors.

[18] Oseev A., Boiseaumarié B.L.R.D., Remy-Martin F., Manceau J.-F., Rouleau A., Chollet F., Boireau W., Leblois T. (2018). Integration of Microresonant Sensor into a Microfluidic Platform for the Real Time Analysis of Platelets-Collagen Interaction in Flow Condition. Proceedings.

[19] Clemetson K.J. (2012). Platelets and Primary Haemostasis. Thromb. Res..

[20] Kroll M., Hellums J., McIntire L., Schafer A., Moake J. (1996). Platelets and shear stress. Blood.

[21] Aoyagi S., Rouleau A., Boireau W. (2008). TOF-SIMS structural characterization of self-assembly monolayer of cytochrome b5 onto gold substrate. Appl. Surf. Sci..

[22] Obeid S., Ceroi A., Mourey G., Saas P., Elie-Caille C., Boireau W. (2017). Development of a NanoBioAnalytical platform for “on-chip” qualification and quantification of platelet-derived microparticles. Biosens. Bioelectron..

[23] Oseev A., Lecompte T.P., Remy-Martin F., Mourey G., Chollet F., Le Roy de Boiseaumarie B., Rouleau A., Bourgeois O., De Maistre E., Elie-Caille C. (2020). Assessment of shear-dependent kinetics of primary haemostasis with a microfluidic acoustic biosensor. IEEE Trans. Biomed. Eng..

[24] Reininger A.J., Heijnen H.F.G., Schumann H., Specht H.M., Schramm W., Ruggeri Z.M. (2006). Mechanism of platelet adhesion to von Willebrand factor and microparticle formation under high shear stress. Blood.

[25] Watson S. (2009). Platelet Activation by Extracellular Matrix Proteins in Haemostasis and Thrombosis. Curr. Pharm. Des..

[26] Sarratt K.L., Chen H., Zutter M.M., Santoro S.A., Hammer D.A., Kahn M.L. (2005). GPVI and α2β1 play independent critical roles during platelet adhesion and aggregate formation to collagen under flow. Blood.

[27] Elie-Caille C., Lascombe I., Péchery A., Bittard H., Fauconnet S. (2020). Molecular and nanoscale evaluation of N-cadherin expression in invasive bladder cancer cells under control conditions or GW501516 exposure. Mol. Cell. Biochem..

[28] Ewald M., Tetard L., Elie-Caille C., Nicod L., Passian A., Bourillot E., Lesniewska E. (2014). From surface to intracellular non-invasive nanoscale study of living cells impairments. Nanotechnology.

[29] Elblbesy M.A., Hereba A.T. (2016). Computation of the Coefficients of the Power law model for Whole Blood and Their Correlation with Blood Parameters. Appl. Phys. Res..

[30] Radmacher M., Fritz M., Kacher C.M., Cleveland J.P., Hansma P.K. (1996). Measuring the viscoelastic properties of human platelets with the atomic force microscope. Biophys. J..

[31] Puchkov E.O. (2013). Intracellular viscosity: Methods of measurement and role in metabolism. Biochem. Suppl. Ser. A Membr. Cell Biol..

[32] Latif U., Can S., Hayden O., Grillberger P., Dickert F.L. (2013). Sauerbrey and anti-Sauerbrey behavioral studies in QCM sensors-Detection of bioanalytes. Sens. Actuators B Chem..

[33] Salomäki M., Kankare J. (2007). Modeling the growth processes of polyelectrolyte multilayers using a quartz crystal resonator. Proc. J. Phys. Chem. B Am. Chem. Soc..

[34] Dultsev F.N., Kolosovsky E.A. (2017). QCM model as a system of two elastically bound weights. Sens. Actuators B Chem..

